# Successful ABO and HLA incompatible kidney transplantation in children in the UK

**DOI:** 10.1007/s00467-022-05583-5

**Published:** 2022-06-13

**Authors:** Eun Yee Hew, Nicos Kessaris, Jelena Stojanovic, Helen Jones, Martin Christian, Anusha Edwards, David V. Milford, Milos Ognjanovic, Mohan Shenoy, Richard J. Baker, Stephen D. Marks

**Affiliations:** 1 NIHR Great Ormond Street Hospital Biomedical Research Centre, University College London Great Ormond Street Institute of Child Health, London, WC1N 1EH UK; 2 Department of Paediatric Nephrology, Great Ormond Street Hospital for Children NHS Foundation Trust, Great Ormond Street, London, WC1N 3JH UK; 3 Department of Nephrology and Transplantation, NHS Foundation Trust, Guy’s and St Thomas, London, SE1 9RT UK; 4 Department of Paediatric Nephro-Urology, Evelina London Children’s Hospital, Westminster Bridge Road, London, SE1 7EH UK; 5 Department of Pediatric Nephrology, Nottingham Children’s Hospital, Nottingham, NG7 2UH UK; 6 Renal Transplantation Unit, Southmead Hospital, Southmead Road, Bristol, BS110 5NB UK; 7 Department of Paediatric Nephrology, Birmingham Children’s Hospital, Steelhouse Lane, Birmingham, B4 6NH UK; 8 Department of Paediatric Nephrology, Great North Children’s Hospital, Royal Victoria Infirmary, Newcastle upon Tyne, NE1 4LP UK; 9 Department of Paediatric Nephrology, Royal Manchester Children’s Hospital, Oxford Road, Manchester, M13 9WL UK; 10 Renal Unit, Lincoln Wing, St. James’s University Hospital, Beckett Street, Leeds, LS9 7TF UK

**Keywords:** ABO-incompatible, HLA-incompatible, Kidney transplantation, Paediatric, Desensitisation

## Abstract

**Background:**

There is increasing evidence of good short-term and medium-term outcomes of ABO incompatible (ABOi) and HLA incompatible (HLAi) kidney transplantation with pre-transplant positive crossmatches in paediatric practice. However, there remain concerns regarding the higher risks of infective complications and antibody-mediated rejections. The aim of our study is to show longer-term follow-up on all ABOi and HLAi paediatric kidney transplant recipients (pKTR) in the UK.

**Methods:**

Questionnaires specifying kidney transplant type, desensitisation requirement and kidney allograft function were sent to 13 paediatric nephrology centres that performed kidney transplantation in children and young people under 18 years of age who received an ABOi and/or HLAi transplant between 1 January 2006 and 31 December 2016. Patient and kidney allograft survival were compared between ABOi, HLAi and ABO/HLA compatible (ABOc/HLAc) groups.

**Results:**

Among 711 living donor kidney transplants performed in the UK, 23 were ABOi and 6 were HLAi. Patient survival was 87%, 100% and 96% in ABOi, HLAi and ABOc/HLAc groups, respectively, at median follow-up of 6.8 (3.6–14.0) years post-transplant. Death-censored kidney allograft survival was 100% in all 3 groups at last follow-up. There were no cases of primary non-function in ABOi or HLAi groups, but 2% in the ABOc/HLAc group. There was one reported case of Epstein-Barr viral-induced post-transplant lymphoproliferative disorder.

**Conclusion:**

Longer term follow-up has shown that ABOi and HLAi kidney transplantation are feasible for pKTR where no compatible donors are available, and that minimising desensitisation should be achieved where possible.

**Graphical abstract:**

A higher resolution version of the Graphical abstract is available as Supplementary information. 
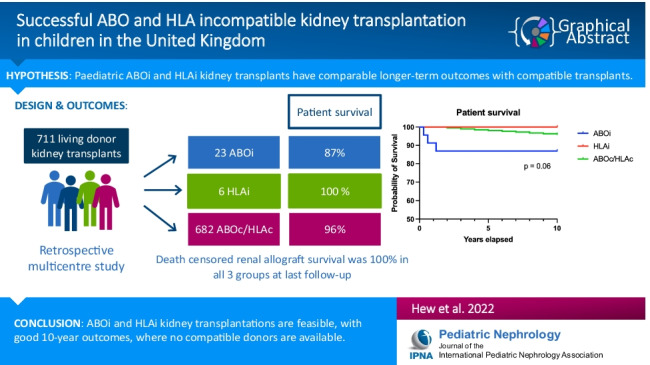

**Supplementary Information:**

The online version contains supplementary material available at 10.1007/s00467-022-05583-5.

## Introduction

It is widely known that successful kidney transplantation is the optimal treatment of choice for children with kidney failure. Besides having detrimental effects on growth and cognitive development [1, 2], children on dialysis have worse outcomes compared to kidney transplant recipients (KTR) [3, 4]. The field of paediatric kidney transplantation has made major advances over the last five decades [5], with marked improvements in patient and kidney allograft survival in recent years [4, 6]. While ABO and HLA compatible kidney transplantation is still preferred, increasing shortage of deceased donor kidneys [7] and sensitisation of children due to blood transfusions and previous transplants [8–10] have resulted in strategies with antibody removal to permit ABO incompatible (ABOi) and HLA incompatible (HLAi) kidney transplantation in children [3, 11–14]. Although there are encouraging and extensive data for adult KTR [15], many centres have been slow to incorporate this method of transplantation into paediatric programmes for several reasons. There still remain concerns that children have a higher risk of acute rejection episodes [16] and infective complications, with the possibility of developing Epstein-Barr viral-induced post-transplant lymphoproliferative disorder (PTLD), especially with the increased burden of immunosuppression [17–19]. To minimise some of the aforementioned risks, tailored desensitisation protocols have been adopted for ABOi and HLAi kidney transplantation [20]. In this paper, we report the longer term outcome for ABOi and HLAi kidney transplantation in children in the UK.

## Patients and methods

Data were obtained from the UK Transplant Registry on all children and young people below the age of 18 years who received a primary living donor paediatric kidney-only transplant between 1 January 2006 and 31 December 2016 from 13 paediatric nephrology centres, and 10 of which perform paediatric kidney transplantation although data were also obtained from adult kidney transplant centres who had performed transplants in adolescents under 18 years of age. There were no records of ABOi or HLAi paediatric living kidney donor transplants prior to 2006. Following transplantation, patient and kidney allograft survival were compared between ABOi, HLAi and ABO/HLA compatible (ABOc/HLAc) groups.

The definition of ABOi kidney transplantation is transplants where the recipient has blood group incompatibility with the donor (e.g. blood group A or B or AB donor into blood group O recipient, or blood group A or AB donor into blood group B recipient, or blood group B or AB donor into blood group A recipient). The definition of HLAi kidney transplantation varies in the literature but we believe this is only where there is a positive crossmatch with positive baseline flow cytometric crossmatch or positive complement dependent cytotoxic crossmatch. Flow cytometry crossmatch is performed on T and B lymphocytes between the patient’s serum and negative control serum. However, where kidney transplantation occurs when the recipient has donor-specific antibody but a negative crossmatch, then this does not fall into the category of HLA incompatible kidney transplantation. Delayed graft function was defined as the requirement for dialysis in the first week post-transplantation [21].

To obtain more detailed information on antibody removal and longer-term follow-up, questionnaires specifying kidney transplant type, desensitisation requirement and kidney allograft function were sent to all 13 paediatric nephrology centres which followed paediatric kidney transplant recipients (pKTR) as well as those adult nephrology centres which had performed kidney transplantation in adolescents under 18 years of age. The last follow-up date included in this study was 31 August 2021. Estimated glomerular filtration rate (eGFR) was calculated using the modified Schwartz formula with plasma creatinine values and heights [22]. Serum creatinine was measured by a modified Jaffe’s kinetic method. A Kaplan–Meier survival curve was plotted showing patient survival. Log-rank test was used to calculate the survival curve (*p* < 0.05 statistically significant).

Results on biopsy-proven acute rejection episodes were obtained where available. Data were collected on complications, including infections and admissions to hospital. Data were fully anonymised and ethical principles were adhered to throughout the study. Data were transferred and handled according to the NHS Blood and Transplant policy; therefore, separate ethical review was not required.

### Desensitisation protocol

The individual desensitisation protocols were personalised depending on the patient and centre as well as known anti-A/anti-B antibody titres and the immune response to treatment prior to transplant [11]. The different desensitisation protocols received by pKTR included pre-transplant antibody removal using immunoadsorption columns for ABOi pKTR with baseline anti-A/anti-B titres greater than 1 in 64. Double filtration plasmapheresis (DFPP), which is a less specific but more cost-effective alternative, was used for patients with baseline titres between 1 in 16 and 1 in 64, as the need for a small number of treatments meant a limited effect on coagulation. The target was to reach anti-A or anti-B antibody titre of 1 in 8 or lower on the day of transplantation.

Pre-operative antibody removal using DFPP or immunoadsorption was generally performed in HLAi pKTR after test plasma exchange with reduction in total MFI from a median of 18,854 (11,602–22,545) to 12,727 (8333–15,321) on the day of transplantation. Donor-specific antibodies for each HLAi patient are listed in Table 1.

**Table 1:** Donor-specific HLA antibodies in HLAi patients

Patient	Donor-specific HLA antibodies
1	DPB1
2	DR14
3	DR17, DR52
4	A23, B8, Cw7, DQ6, DP1
5	B7, DQ8
6	Not reported

Individual desensitisation protocols for both ABOi and HLAi groups are summarised in Table 2.

**Table 2:** Desensitisation protocols received by pKTR across ABOi, HLAi and ABOc/HLAc

	ABOi	HLAi
Rituximab (R)	4	
Intravenous Immunoglobulin (IVIg)		1
Plasmapheresis (P)		
Double filtration plasmapheresis (DFPP)	2	
Immunoadsorption (I)	3	
2 different desensitisation regimens		
- DFPP + IVIg		1
- R + P		1
- R + DFPP	2	
- R + I	1	
Not reported	11	3
Total	23	6

### Immunosuppression regimens

For the ABOi group, pre-transplant intravenous rituximab was usually given one month prior to kidney transplantation when the baseline titre was above 1 in 8. Intravenous basiliximab induction treatment was used on day 0 and 4 post-kidney transplantation in 22 ABOi pKTR with one patient receiving alemtuzumab peri-operatively. Either anti-thymocyte globulin, alemtuzumab or basiliximab was used for induction immunosuppression for all HLAi pKTR (Table 3).

**Table 3:** Baseline patient characteristics (data shown are median and interquartile range or %)

	ABOi	HLAi	ABOc/HLAc
Age (year) (median, IQR)	10.3 (6.2–14.1)	12.8 (9.7–13.7)	10.9 (5.8–15.0)
Sex (Male (%))	13 (57%)	4 (67%)	477 (70%)
Kidney disease			
CAKUT (dysplasia ± PUV ± VUR) FSGS CNS WT/DDS RVT HUS Unknown	39% 9% 13% 17% 9% 9% 4%	67% 17% 17% 0% 0% 0% 0%	63% 12% 8% 6% 6% 1% 4%
Number of previous kidney transplants			
1 2	18/23 (78%) 5/23 (22%)	0/6 (0%) 6/6 (100%)	560/682 (82%) 122/682 (18%)
Modality of kidney replacement therapy at time of transplant			
Peritoneal dialysis Haemodialysis Pre-emptive kidney transplant	7/23 (30%) 8/23 (35%) 8/23 (35%)	1/6 (17%) 3/6 (50%) 2/6 (33%)	302/682 (44%) 140/682 (21%) 240/682 (35%)
Induction treatment			
Alemtuzumab Basiliximab Anti-thymocyte globulin	1/23 (4%) 22/23 (96%) 0/23 (0%)	4/6 (67%) 1/6 (17%) 1/6 (17%)	0/682 (0%) 162/682 (24%) 0/682 (0%)

*PUV*, posterior urethral valve; VUR, vesicoureteral reflux; FSGS, focal segmental glomerulosclerosis; *CNS*, congenital nephrotic syndrome; WT/DDS, Wilm’s tumour/Denys-Drash syndrome; *RVT*, renal venous thrombosis; *HUS*, haemolytic uraemic syndrome

Corticosteroids, mycophenolate mofetil (MMF) and tacrolimus were the most common combination for maintenance immunosuppression in ABOi and HLAi, while corticosteroids, azathioprine and tacrolimus were the most common in the ABOc/HLAc group (Table 4).

**Table 4:** Immunosuppression at three months following primary living paediatric kidney only transplantation

Immunosuppression	ABOi	HLAi	ABOc and HLAc	Total
SAT	2 (10%)	0 (0%)	194 (31%)	196
SMT	14 (70%)	5 (83%)	174 (28%)	193
MT	2 (10%)	1 (17%)	150 (24%)	153
Other	2 (10%)	0 (0%)	109 (17%)	111
Not reported	3	0	55	58
Total	23	6	682	711

*S*, steroid; *A*, azathioprine; *M*, mycophenolate; *T*, tacrolimus

## Results

A total of 711 living donor kidney transplants were performed in the UK in children and young people under 18 years from 1 January 2006 to 31 December 2016, of which 23 were ABOi and 6 were HLAi. There were 682 ABOc/HLAc kidney transplants performed and no cases of combined ABOi and HLAi transplants in this cohort. There was no difference in pre-emptive transplantation rates (35%, 33% and 35% in ABOi, HLAi and ABOc/HLAc groups (Table 3).

Patient survival was 87%, 100% and 96% in ABOi, HLAi and ABOc/HLAc groups, respectively, at median follow-up of 6.8 (3.6–14.0) years post-transplant (Table 5). The causes of death of the three patients in the ABOi group were unknown in the first patient who had an undiagnosed syndrome and unexpectedly died overnight 3 months post-transplant without post-mortem being performed, the second patient had Epstein-Barr viral-induced PTLD at 7 months post-transplant, and the third patient had gastrointestinal disease unrelated to the kidney transplant at 14 months post-transplant.

**Table 5:** Patient survival at median follow-up of 6.8 (3.6–14.0) years post-transplant

	ABOi	HLAi	ABOc/HLAc
Survived	20 (87%)	6 (100%)	655 (96%)
Did not survive	3 (13%)	0 (0%)	27 (4%)
Total	23	6	682

**Fig. 1:**
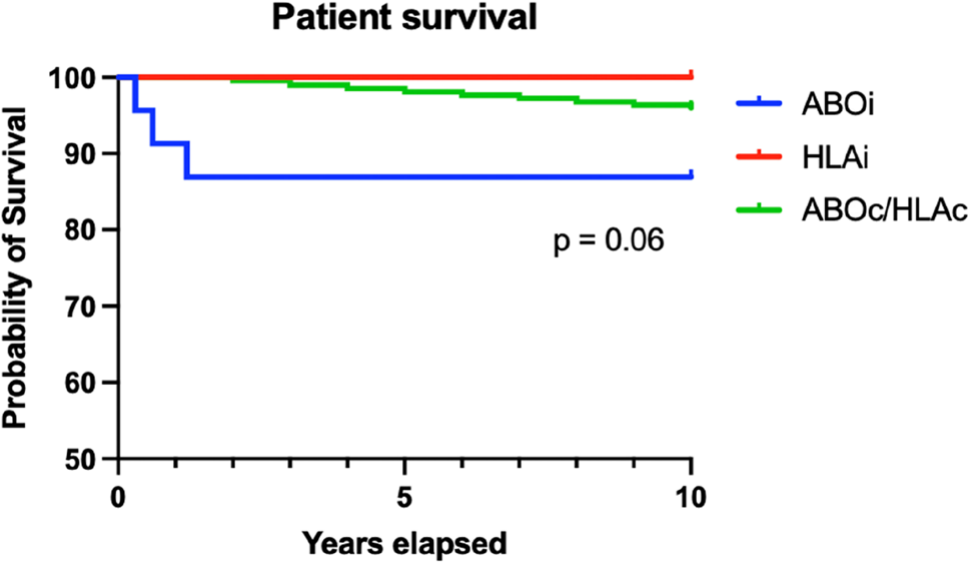
Kaplan–Meier survival curve comparing patient survival between ABOi, HLAi and ABOc/HLAc groups

A Kaplan–Meier survival curve was plotted comparing patient survival between ABOi, HLAi and ABOc/HLAc (Fig. 1). There was no statistically significant difference between the three groups (log-rank test, *p* = 0.06).

Death-censored kidney allograft survival was 100% in all groups (ABOi, HLAi and ABOc/HLAc) at last follow-up. The rate of delayed graft function was 6%, 0% and 3% in the ABOi, HLAi and ABOc/HLAc groups, respectively. There were no cases of primary non-function in the ABOi or HLAi groups, but 2% in the ABOc/HLAc group. The median eGFR was 62, 76 and 101 ml/min/1.73 m^2^ in the ABOi, HLAi and ABOc/HLAc groups at last follow-up (Table 6).

**Table 6:** Renal allograft function following primary living paediatric kidney only transplantation (eGFR shown as median and interquartile range)

Graft function	ABOi	HLAi	ABOc and HLAc	Total
Immediate	17 (94%)	6 (100%)	578 (94%)	601
Delayed	1 (6%)	0 (0%)	20 (3%)	21
Primary non-function	0 (0%)	0 (0%)	15 (2%)	15
Not reported	5	0	69	74
eGFR (ml/min/1.73 m^2^)	62 (46–75)	76 (61–101)	101 (74–144)	
Total	23	6	682	711

### Biopsy proven acute rejections

In all centres, for-cause biopsies were mainly carried out in the ABOi group whereas surveillance biopsies were mainly performed in the HLAi group. In the ABOi group, 22 biopsies were performed in 16 pKTR with four episodes (18.2%) of biopsy proven acute T-cell-mediated rejection within the first 12 months after transplantation. In the HLAi group, 16 biopsies were performed in 5 pKTR with no biopsy proven acute rejection episodes (0%).

### Infectious complications

In this study, criteria of viraemia are defined as the detection of PCR DNA of respective viruses in plasma and blood samples according to local protocols. Ten viraemic events occurred in the ABOi group in the first 12 months post-transplant: 4 with cytomegalovirus (CMV), 3 with Epstein-Barr virus (EBV) and 3 with BK virus, while nine viraemic events occurred in the HLAi group: 1 with CMV, 3 with EBV, 3 with BK and 2 reported with JC virus.

Of these, 2 ABOi and 3 HLAi patients had more than one viraemia. Among ABOi patients, one pKTR had evidence of both CMV and BK viraemia; another had evidence of both CMV and EBV viraemia. Meanwhile, among HLAi patients, 2 pKTR had evidence of BK, JC and EBV viraemia, and another had CMV and BK viraemia.

Among 682 ABOc/HLAc patients, 55 had viraemia with 30, 15 and 16 cases of EBV, CMV and BK viraemia, respectively. Of these, two pKTR had evidence of both CMV and EBV viraemia; another two had evidence of CMV and BK viraemia; and another two pKTR had evidence of EBV and BK viraemia although there is likely underreporting in these patients (Table 7).

**Table 7:** Infection complications in the first 12 months post-transplant of ABOi, HLAi and ABOc/HLAc

	ABOi	HLAi	ABOc/HLAc
Cytomegalovirus (CMV)	4	1	15
Epstein-Barr virus (EBV)	3	3	30
BK virus (BKV)	3	3	16
JC virus (JCV)		2	
More than 1 viraemia			
- CMV + BKV	1		2
- CMV + EBV	1		2
- EBK + BKV			2
- BKV + JCV + EBV		2	

Total viraemic events in each group: ABOi — 10, HLAi — 9, ABOc/HLAc — 55

Four patients developed urinary tract infections (UTIs), of which 3 were diagnosed based on clinical symptoms and 1 developed *Escherichia coli* urosepsis (defined as presence of SIRS criteria and positive urine cultures). There was one reported case of Epstein-Barr viral-induced PTLD in the ABOi cohort.

## Discussion

This is the first report in the literature of longer term outcomes from a large paediatric ABOi and HLAi kidney transplantation national programme with comparable longer term outcomes with compatible transplants. Our study has shown that patient survival was similar across ABOi, HLAi and ABOc/HLAc groups at 87%, 100% and 96% in ABOi, HLAi and ABOc/HLAc groups, respectively, at median follow-up of 6.8 (3.6–14.0) years post-transplant with 100% death censored kidney allograft survival in all three groups. There were no cases of primary non-function in the ABOi or HLAi groups, but 2% in the ABOc/HLAc group. However, it should be noted that there was one reported case of Epstein-Barr viral-induced PTLD in the ABOi kidney transplantation group.

With the shortage of deceased donor kidneys and sensitisation of children due to blood transfusions and previous transplants, ABOi and HLAi kidney transplantation has been performed more often in the last few decades [13, 14, 23, 24]. There is increasing evidence in the literature that kidney transplantation offers significant survival advantages compared to remaining on dialysis [4, 25]. We previously reported excellent outcomes with kidney allograft survival and acute rejection rates in 11 paediatric ABOi kidney transplant recipients using tailored desensitisation protocols [11]. Meanwhile, in adults, Massie et al. also reported higher cumulative survival at 5 and 10 years compared to similar patients who remained on the waitlist to potentially receive an ABO compatible kidney transplant [26].

Unfortunately, wait times are long for highly sensitised patients [27, 28]. In the UK, a national living donor kidney sharing scheme exists and incompatible pairs are advised to enter the scheme. Ideally, there would be a trial of at least three quarterly runs although successful matching runs are less likely in highly sensitised children [13]. For highly sensitised patients, positive cross-matched transplantation after appropriate desensitisation may lead to better patient survival than staying on dialysis [3, 11, 29]. The living donor pool can be significantly expanded through this approach.

Our study has shown that there was no statistically significant difference (*p* = 0.06) in patient survival between the ABOi, HLAi and ABOc/HLAc groups, and death-censored kidney allograft survival was 100% in all three groups. Although the *p*-value is 0.06 with more patient deaths in the ABOi group, due to the small sample size, it is likely that one child who died because of their underlying syndrome, another child who died of a gastrointestinal issue not related to their transplant and one case of PTLD is not significant. There were also no cases of primary non-function in the ABOi or HLAi groups.

For-cause biopsies were mainly carried out in the ABOi group whereas surveillance biopsies were mainly performed in the HLAi group. In the ABOi group, 22 biopsies were performed in 16 pKTR with four episodes (18.2%) of biopsy proven acute T-cell-mediated rejection within the first 12 months after transplantation, while 0% of HLAi developed acute rejection episodes. These results could be due to increased immunosuppression in the HLAi group, but a greater sample size would be required to demonstrate if there is any significant difference.

There were potential limitations in our study. The baseline characteristics were different among the three groups. The sample sizes for the ABOi and HLAi groups were small compared to the ABOc/HLAc group, where there could be underreporting of complications. The interpretation of the results was also limited by the retrospective nature and the varying protocols for desensitisations and patient monitoring used across the centres.

Therefore, for future studies, we would recommend a larger sample size with further longer term follow-up of ABOi, HLAi and ABOc/HLAc pKTR with the development of national programmes supporting specialised care. An extensive and continuous collaboration with other paediatric kidney transplant centres is also needed to further investigate the outcomes for ABOi and HLAi pKTR.

In conclusion, our study has shown that ABOi and HLAi kidney transplantations are feasible for pKTR, with good 10-year outcomes, where no compatible donors are available.

## Supplementary Information

Below is the link to the electronic supplementary material.

Graphical Abstract (PPTX 232 kb)
