# Supplementary material for: Successful ABO and HLA incompatible kidney transplantation in children in the UK
Source: Pediatr Nephrol. 2022 Jun 13;38(2):529–35. doi: 10.1007/s00467-022-05583-5 (PMC9763153; doi:10.1007/s00467-022-05583-5)
Supplement: Supplementary file 1 — Graphical Abstract (PPTX 232 kb) [file 467_2022_5583_MOESM1_ESM.pptx]

## Slide 1
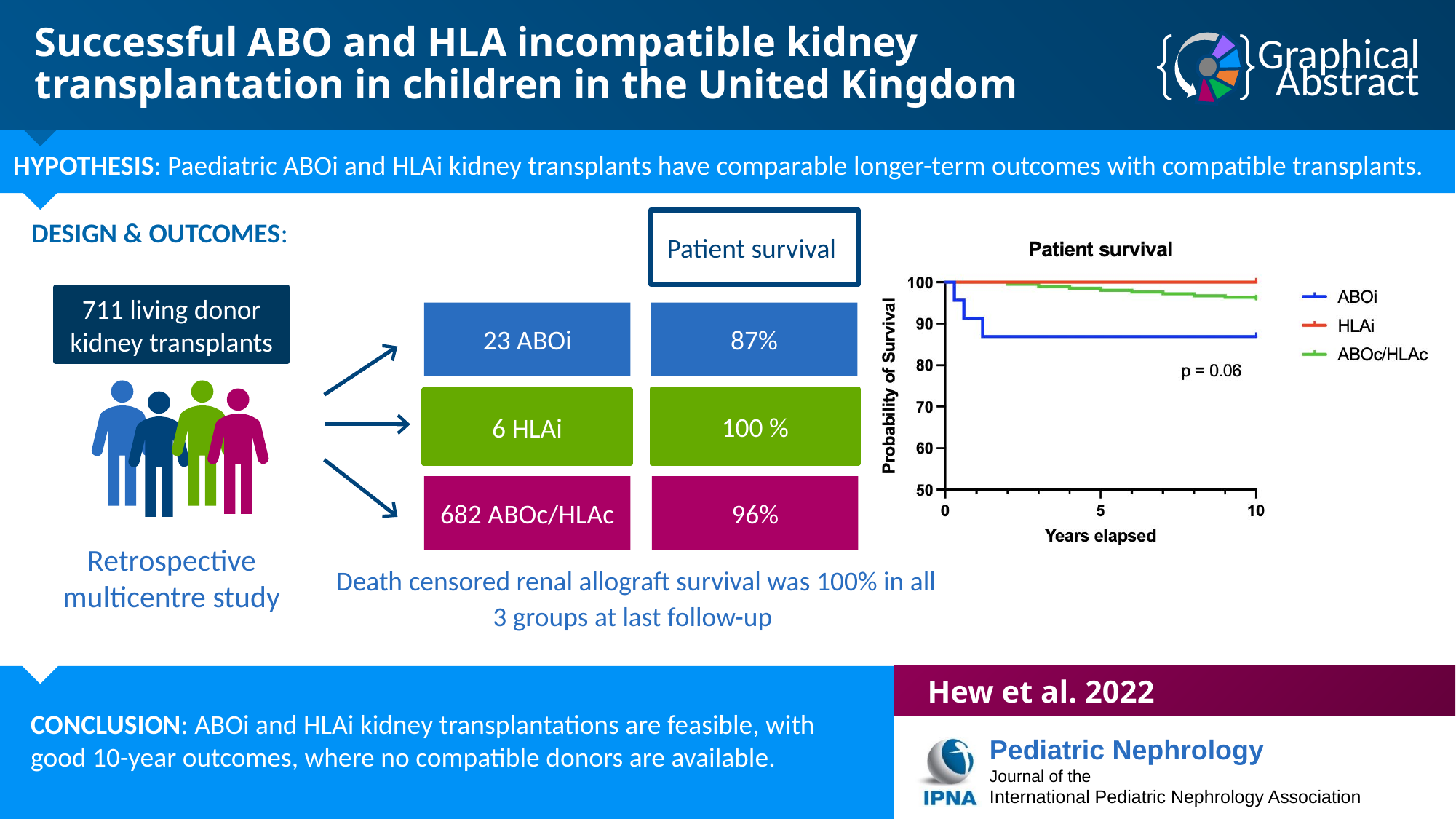

Successful ABO and HLA incompatible kidney transplantation in children in the United Kingdom
HYPOTHESIS: Paediatric ABOi and HLAi kidney transplants have comparable longer-term outcomes with compatible transplants.
DESIGN & OUTCOMES:
Patient survival
711 living donor kidney transplants
23 ABOi
87%
100 %
6 HLAi
96%
682 ABOc/HLAc
Retrospective multicentre study
Death censored renal allograft survival was 100% in all 3 groups at last follow-up
Hew et al. 2022
CONCLUSION: ABOi and HLAi kidney transplantations are feasible, with good 10-year outcomes, where no compatible donors are available.
